# Identification of Risk Factors and Development of Predictive Risk Score Model for Mortality after Open Ruptured Abdominal Aortic Aneurysm Repair

**DOI:** 10.3390/medicina58040549

**Published:** 2022-04-15

**Authors:** Ivan Tomic, Petar Zlatanovic, Miroslav Markovic, Milos Sladojevic, Perica Mutavdzic, Ranko Trailovic, Ksenija Jovanovic, David Matejevic, Biljana Milicic, Lazar Davidovic

**Affiliations:** 1 Faculty of Medicine, University of Belgrade, 11000 Belgrade, Serbia; drmiroslav@gmail.com (M.M.); milos.sladojevic@gmail.com (M.S.); mutavdzic_perica@yahoo.com (P.M.); rankotrailovic@yahoo.com (R.T.); ksenia.stevanovic@gmail.com (K.J.); davidovic.lazar@gmail.com (L.D.); 2 Clinic for Vascular and Endovascular Surgery, Clinical Centre of Serbia, 11000 Belgrade, Serbia; petar91goldy@gmail.com (P.Z.); davidmatejevic@hotmail.com (D.M.); 3 Department for Statistics and Informatics, Faculty of Dental Medicine, University of Belgrade, 11000 Belgrade, Serbia; milicic.biljana85@gmail.com

**Keywords:** ruptured abdominal aortic aneurysm (RAAA), mortality, risk score, prediction

## Abstract

*Background and Objectives:* Despite the relatively large number of publications concerning the validation of these models, there is currently no solid evidence that they can be used with absolute precision to predict survival. The goal of this study is to identify preoperative factors that influenced 30-day mortality and to create a predictive model after open ruptured abdominal aortic aneurysm (RAAA) repair. *Materials and Methods:* This was a retrospective single-center cohort study derived from a prospective collected database, between 1 January 2009 and 2016. Multivariate logistic regression analysis was used to identify all significant predictive factors. Variables that were identified in the multivariate analysis were dichotomized at standard levels, and logistic regression was used for the analysis. To ensure that dichotomized variables were not overly simplistic, the C statistic was evaluated for both dichotomized and continuous models. *Results:* There were 500 patients with complete medical data included in the analysis during the study period. Of them, 37.6% were older than 74 years, and 83.8% were males. Multivariable logistic regression showed five variables that were predictive of mortality: age > 74 years (OR = 4.01, 95%CI 2.43–6.26), loss of consciousness (OR = 2.21, 95%CI 1.11–4.40), previous myocardial infarction (OR = 2.35, 95%CI 1.19–4.63), development of ventricular arrhythmia (OR = 4.54, 95%CI 1.75–11.78), and DAP < 60 mmHg (OR = 2.32, 95%CI 1.17–4.62). Assigning 1 point for each variable, patients were stratified according to the preoperative RAAA mortality risk score (range 0–5). Patients with 1 point suffered 15.3% mortality and 3 points 68.2% mortality, while all patients with 5 points died. *Conclusions:* This preoperative RAAA score identified risk factors readily assessed at the bedside and provides an accurate prediction of 30-day mortality after open repair of RAAA.

## 1. Introduction

Despite the constant progress of surgical techniques and technological innovations, mortality after open surgical treatment of ruptured abdominal aortic aneurysm (RAAA) has remained unchanged for decades [1]. In recent years, the results of several randomized multicenter studies speak in favor of declining mortality trends, which many attribute to the introduction and widespread use of endovascular treatment (EVAR) [2,3]. EVAR is still not possible perform in 40–65% of RAAA patients, both due to anatomical limitations and the unavailability of adequate endovascular stent grafts in emergency settings, especially in low- and medium-income countries [4]. The final outcome and quality of treatment is the product of various pre-, intraoperative, but also factors specific to the center, such as annual and cumulative volume of the patients and its specialization for the treatment of RAAA.

Today, there is a growing record of conservative treatment of RAAA. According to available publications, the average proportion of untreated patients ranges between 21.8% in the United States and 48.8% in the United Kingdom, while a meta-analysis observing patients treated in Finland, Sweden, Denmark, the United Kingdom, Australia, and New Zealand identified around 40% of RAAA patients undergoing palliation [5,6,7].

Many surgeons refer to some of the available predictive models during the decision-making process. Despite the relatively large number of publications concerning the validation of these models, there is currently no solid evidence that they can be used with absolute precision to predict survival [8,9]. Current European Society for Vascular Surgery (ESVS) Guidelines for the management of aortoiliac aneurysms do not recommend the use of existing scoring systems for decision making [10]. However, the scoring systems might be of importance when we keep in mind the high cost of treatment due to frequent and severe postoperative complications.

Taking into account the large volume of patients operated on due to RAAA [11], the goal of this study is to identify preoperative factors that influence 30-day mortality, as well as to create a predictive model after open RAAA repair. 

## 2. Methods

### 2.1. Patient Population and Study Design

This was a retrospective single-center cohort study derived from a prospective collected database of patients who were operated on at the Clinic for Vascular and Endovascular Surgery at the University Clinical Centre of Serbia between 1 January 2009 and 1 January 2016. All consecutive patients older than 18 with confirmed diagnosis of RAAA that were operated on and with complete medical data were included in our study. Diagnosis of RAAA was confirmed using one of the following imaging studies: duplex ultrasound (US), conventional angiography, and computerized angiography (CTA).

This study excluded patients with symptomatic, mycotic AAA and patients with thoracoabdominal aortic aneurysms (ThAAA). Informed consent was obtained from all conscious individuals included in this study. For unconscious patients, we acted in accordance with the legal regulations in our country, that medical data of patients treated in university clinics can be anonymously used for scientific research purposes without special consent. Patients who survived gave us a signature after the operation. The study was approved by the local committee at the Clinical Centre of Serbia. Only attending physicians were allowed to assess the non-anonymized data. Data were collected in accordance with the Declaration of Helsinki, and the study was carried out in accordance with the STROBE statement [12].

### 2.2. Data Collection

Although the study included 109 variables, we decided to focus our analysis on those easily acquired variables in the preoperative setting:

- Demographics: age and gender
- Baseline comorbidities: smoking status, obesity, presence of hypertension, diabetes mellitus, previous myocardial infarction, previous myocardial revascularization, angina pectoris, heart failure (defined as ejection fraction less than 40%), chronic obstructive pulmonary disease (COPD), chronic kidney disease (CKD), cerebrovascular disease (previous stroke/transient ischemic attack (TIA)), atrial fibrillation
- Preoperative laboratory: blood count, renal function analysis
- Clinical presentation: systolic, diastolic/mean arterial blood pressure (SAP/DAP/MAP), preoperative shock defined as MAP less than 60 mmHg lasting more than 20 min, state of consciousness, duration of symptoms onset until the surgery, presence of abdominal/lower back pain, presence of collapse, development of ventricular arrhythmia, and cardiac arrest.
- Furthermore, to describe the study group, additional intraoperative and immediate postoperative data were presented as well:
- RAAA parameters: maximal AAA diameter, AAA location (infrarenal, juxtarenal, pararenal, iliac artery aneurysm).
- Intraoperative data: site of the proximal clamp and proximal clamping duration, type of reconstruction, total operative time, intraoperative blood loss, and number of given blood transfusion units.
- Postoperative data/complications: acute kidney injury, pulmonary complications (prolonged ventilation for more than 72 h, pneumonia, atelectasis), surgical reintervention, major bleeding requiring reintervention, lower limb ischemia, stroke, acute coronary syndrome, ischemic colitis, sepsis, wound infection, wound dehiscence, abdominal compartment syndrome.

### 2.3. Endpoints and Statistical Analysis

The primary endpoint was immediate 30-day mortality.

To assess normal distributions, we used the Shapiro–Wilk test. All results were expressed as arithmetic mean (X) ± standard deviation (SD) for normally distributed variables and as median and lower and upper interquartile ranges for non-normally distributed variables. Categorical variables were presented as absolute and relative frequencies. Differences in continuous variables between the groups were analyzed by Student’s *t*-test or by Mann–Whitney U test for variables with skewed distributions.

First univariate and then multivariate logistic regression analysis for 30-day mortality was used to identify all significant predictive factors. To achieve a practical risk score, variables identified on multivariate analysis were dichotomized at standard levels, and logistic regression was used for the analysis. To ensure that dichotomized variables were not overly simplistic, the C statistic was evaluated for both dichotomized and continuous models, and the performance of the two models was comparable. Using the receiver operating characteristic (ROC) curve, the percentage of correctly classified patients was calculated. The accuracy of the model was tested by calculating the area under the curve (AUC).

## 3. Results

There were 530 patients who presented to our center between 1 January 2009 and 1 January 2016. Of these, 500 patients with complete medical data were included in our study. Three patients of total RAAA number underwent palliative treatment (two patients because of terminal phase of malignancy, one patient rejected operation after admission). Twenty-seven (5.1%) patients were excluded from this study because of missing important medical data. All patients underwent consecutive open repair, since at that time we did not have readily available stent grafts in an emergent setting. The baseline clinical characteristics and laboratory for the whole study group are shown in Table 1. The majority of patients were younger than 74 years (63.4%), male (83.8%), smokers (57.4%), and had hypertension (92.2%). In addition, the majority of patients presented with abdominal/lower back pain (99%), pulsatile abdominal mass (87.6%), hypovolemic shock (73.8%), and DAP lower than 60 mmHg (58.6%).

A comparison of baseline clinical and laboratory characteristics between patients based on 30-day mortality is shown in Table 1. Patients who died were older (60.5% vs. 25.1%, *p* < 0.001) and more frequently were female (21.5% vs. 13.3%, *p* = 0.022), had a previous myocardial infarction (26.6% vs. 13.9%, *p* < 0.001), had angina pectoris (49.2% vs. 24.5%, *p* < 0.001), were in heart failure (29.9% vs. 10.8%, *p* < 0.001), were in atrial fibrillation (17.5% vs. 9%, *p* = 0.005), had a previous stroke/TIA, had COPD (48% vs. 29.7%, *p* < 0.001), or had CKD (19.8% vs. 11.1%, *p* = 0.008). Regarding the clinical presentation, patients who died more often had hypovolemic shock (90.4% vs. 64.7%, *p* < 0.001), collapse (67.2% vs. 37.5%, *p* < 0.001), unconsciousness (36.2% vs. 9.6%, *p* < 0.001), and developed ventricular arrhythmia (14.1% vs. 3.7%, *p* < 0.001), as well as cardiac arrest (5.1% vs. 0.3%, *p* < 0.001). Additionally, patients who died had worse hemodynamic parameters, i.e., more patients with SAP < 70 mmHg (42.4% vs. 13.6%, *p* < 0.001), those with DAP < 60 mmHg (80.2% vs. 46.7%, *p* < 0.001) and with MAP < 60 mmHg (56.5% vs. 26.9%, *p* < 0.001). Laboratory parameters were also worse in patients who died, i.e., more patients with hemoglobin < 78 g/L (39% vs. 15.2%, *p* < 0.001), platelets < 145 × 10^9^ (52.5% vs. 26.9%, *p* < 0.001) and creatinine > 145 mmol/L (46.9% vs. 23.5%, *p* < 0.001).

The operative details are shown in Table 2. Most of the AAA were infrarenal (81.8%). The most commonly used clamp site for proximal bleeding control was supraceliac (67.8%), while the mean duration of proximal clamp was 36.6 ± 24.5 min. The most commonly performed reconstruction was tube graft interposition (52.4%). The mean operative time was 166.5 min, while the median blood loss was 3000 mL.

Postoperative complications are shown in Table 3. Pulmonary complications were the most common (22.2%), followed by acute kidney injury (14%) and surgical reintervention (13.8%).

**Figure 1:**
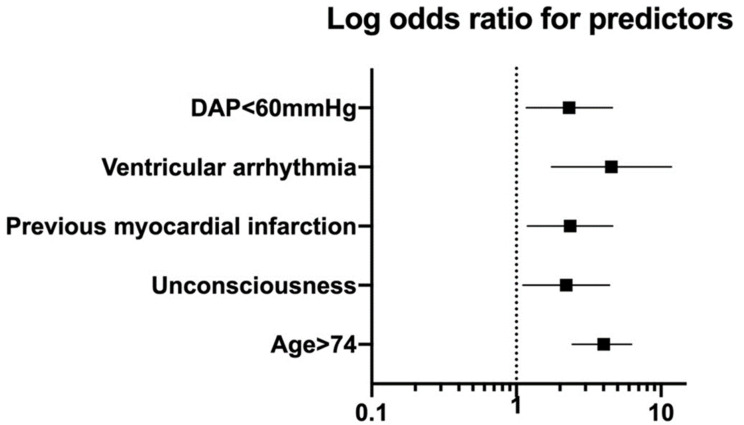
Multivariate logistic regression predictors of 30-day mortality in a study cohort.

Overall, 30-day mortality was 35.4%. Multivariable logistic regression showed five variables that were predictive of mortality: age > 74 years (OR = 4.01, 95%CI 2.43–6.26, *p* < 0.001), loss of consciousness (OR = 2.21, 95%CI 1.11–4.40, *p* = 0.024), previous myocardial infarction (OR = 2.35, 95%CI 1.19–4.63, *p* = 0.014), development of ventricular arrhythmia (OR = 4.54, 95%CI 1.75–11.78, *p* = 0.002), and DAP < 60 mmHg (OR = 2.32, 95%CI 1.17–4.62, *p* = 0.016) (Table 4 and Figure 1).

**Figure 2:**
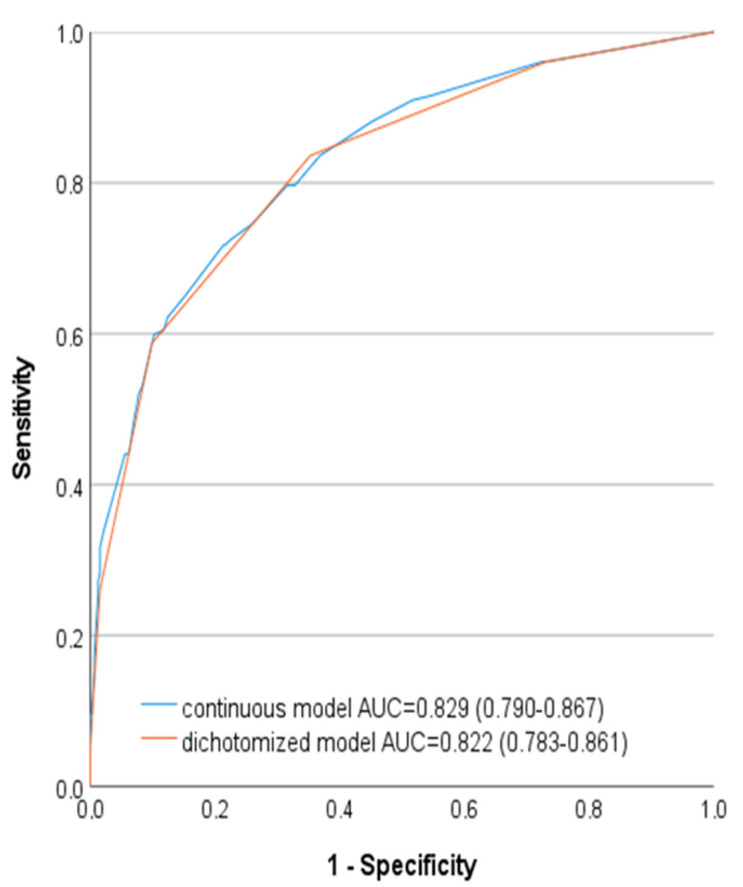
Comparison of two models with continuous and dichotomized variables.

Our score for preoperative RAAA mortality was calculated by assigning 1 point for each of the predictors of 30-day mortality (Table 5). Table 6 shows the distribution of patients according to the preoperative RAAA mortality risk score. Score 1 predicted only 15.3%, score 3 predicted 68.2% 30-day mortality, and score 5 predicted 100% mortality (Table 6). ROC curve (Figure 2) showed almost identical performance of dichotomized and continuous models.

## 4. Discussion

In this research, we sought to identify significant preoperative factors that influenced 30-day mortality after RAAA and to create a predictive risk score in patients undergoing open repair. Five important predictive factors were identified: age > 74 years, previous myocardial infarction, loss of consciousness, development of ventricular arrhythmia, and DAP values < 60 mmHg. With an increase in predictive factors, mortality rose; the presence of all five factors had a 100% prediction of mortality (Table 6).

Over the last 20 years, numerous predicting scoring systems have been developed to predict survival after RAAA (13–19). One of the oldest and most accepted scores is the Hardman index (HI) and Glasgow Aneurysm Score (GAS) [13,14]. However, the predictive value of HI and GAS is low in the highest risk population. In addition, these older models [13,14,15] are not adopted for endovascular repair. The lack of newer predictive models [16,17,18,19] is the absence of external validation, as well as the involvement of intraoperative factors [17]. However, in the current era of high-value and quality care, the goal of a useful and practical scoring system is to predict 30-day mortality that might help in guiding a discussion with the patient and family, which might help with the decision to select between palliative care or going with the patient to the operating theatre. Good prediction systems should be easy to handle and calculate in everyday settings based on preoperative data. Taking into account the big experience of the Clinic for Vascular and Endovascular Surgery [11], we sought to create a new predictive score that would be easier for calculation and applicable than the existing scoring systems.

It is not surprising at all for us to see the existing predictors. Older age (>74 years) is already marked in all existing prediction models, since this represents the marker of organism frailty. Previous myocardial infarction indicates the presence of vulnerable myocardium, while the development of ventricular arrhythmia points to the lack of myocardial perfusion and suffering due to hypovolemic state and hemoglobin depletion, which decrease the blood oxygen caring capacity. Loss of consciousness and lower DAP values (>60 mmHg) simply show the severity of hypovolemic shock. The value of our score is that it is simple and uses only preoperative variables that are readily measured in minutes at the bedside. Other good points of our score are the high prediction of mortality and identical performance of the continuous and dichotomized models, as shown in Figure 1. The degree of overlap of the dichotomized and continuous models allowed us to assign one point for each predictor and resulted in the creation of a simple formula.

Mortality prediction after RAAA repair is important for several reasons. One of the most important reasons is the ability to plan patient care. It is well known that care centralization for RAAA surgical treatment allows the achievement of better results. Patients treated in high volume centers, university hospitals, or specialized trauma centers have a better chance for survival [1,20]. Additionally, the rejection rate is lower in high-volume and teaching hospitals [6]. Better results are conditioned by the availability of a multidisciplinary and well-trained team, as well as by developed diagnostic and therapeutic algorithms adapted to local conditions [11]. The possibility of rapid transport to the reference hospital, resuscitation, and fast transfer to the operation room does not necessarily mean that we have made a good decision for the patient. The key question for physicians in such urgent circumstances is how to identify a patient who cannot be helped. From the other point of view, the question is whether the RAAA repair will enable the patient’s life to be prolonged and what the impact will be on life quality. Despite the technological and organizational progress in RAAA surgery (improvement of treatment protocols, higher contribution of endovascular repair and care centralization), there are still patients who are dying because of RAAA [21,22,23]. The importance of 30-day mortality prediction before a patient’s transfer to a tertiary hospital is to help allocate resources and spare the patient as well physician from performing the procedure with no prospect of success. In addition to the listed reasons, the high cost of RAAA treatment should be taken into account. These costs are often higher than the costs for electively treated patients. This is due to the high rate of complications and prolonged stay in intensive care units. The cost analysis of surgery for RAAA published by authors from the United Kingdom showed that median cost for uncomplicated ruptures was £6427, £20.075 for complicated ruptures, and £4762 for unruptured AAA [24]. Despite this, there are no precise cost data for Serbia. Considering the lower available budget compared to most developed countries, rationalization in the treatment of RAAA patients with higher preoperative scores can be very important.

Predictors that have included in our score are all data that the referring physician who first comes into contact with an RAAA patient can easily communicate with a reference hospital. With data that are available in Table 6, the physicians now have the data to tell the patient and the patients’ families that transfer may be futile when ≥4 risk factors are present. As we have patients who are traveling as far as 300 km from the south of Serbia, the option of not operating on a patient with an 88.1% to 100% likelihood of mortality allows the family to spend time with the patient and initiate comfort care measures. Additionally, the costs of transport from these hospitals are no less important.

Creating simple and reliable preoperative predictive scores can be used in the future to create an application for smartphones in collaboration with information technology experts. The existence of the application can facilitate and ease everyday use of the proposed score.

## 5. Limitations and Strengths

Our study has several limitations. First, this was a single-center study. Second, our score is reflective of our institution and has not been validated at other institutions. Whereas the recognition of RAAA with the need to have a “rupture protocol” has been extremely successful at our hospital, it may not be applicable to other low-volume centers. Third, the majority of our patients were transferred from an outside hospital, and the mere fact that they survived transport might have biased the results. Fourth, all of our patients underwent open repair, which might limit the applicability of this score in patients undergoing endovascular repair. Fifth, only patients who received surgical intervention were included in the present study, and patients who chose conservative treatment, as well as those who died on their way to the hospital, were not included. Sixth, our study did not report the rate of mortality for RAAA at 90 days in order to detect all the deaths related to the index procedures. Seventh, twenty-seven (5.1%) patients were excluded from this study because of missing important medical data.

The main strength of this study is the large number of patients with detailed clinical information, which provides the statistical power to create a strong prediction score. Second, all of these patients were treated at a single institution that has a high volume for aortic pathology, and all our data were obtained from this population and not saturated with results from other institutions, allowing us to believe strongly that this model works at our institution. Third, all of the patients were treated using the same protocol that was developed in 2002 for these patients, and by the same surgical team that uses the same technique and principles when treating these patients [11].

## 6. Conclusions

This preoperative RAAA score identified risk factors readily assessed at the bedside and provides an accurate prediction of 30-day mortality after open repair of RAAA. These findings and results might have a direct impact on decision-making, such as patient referral to a tertiary care center and helping in the discussion with family and relatives.

## Figures and Tables

**Table 1:** Baseline clinical characteristics and comparison between the two groups of patients.

	*n* = 500	*n* = 177	*n* = 323	
Demographics	Whole Study Group	Patients Who Died	Patients Who Survived	*p* Value
Age > 74	188 (37.6%)	107 (60.5%)	81 (25.1%)	<0.001
Female	81 (16.2%)	38 (21.5%)	43 (13.3%)	0.022
**Risk factors**				
Obesity	176 (35.2%)	71 (40.1%)	105 (32.5%)	0.08
Smoking	287 (57.4%)	103 (58.5%)	184 (57.1%)	0.76
Hypertension	461 (92.2%)	166 (93.8%)	295 (91.3%)	0.33
Diabetes mellitus	84 (16.8%)	34 (19.2%)	50 (15.5%)	0.28
Previous myocardial infarction	92 (18.4%)	47 (26.6%)	45 (13.9%)	<0.001
Previous myocardial revascularisation	46 (9.2%)	20 (11.3%)	26 (8%)	0.23
Angina pectoris	60 (12%)	87 (49.2%)	79 (24.5%)	<0.001
Heart failure	88 (17.6%)	53 (29.9%)	35 (10.8%)	<0.001
Atrial fibrillation	60 (12%)	31 (17.5%)	29 (9%)	0.005
Previous stroke	47 (9.4%)	24 (13.6%)	23 (7.1%)	0.018
COPD	181 (36.2%)	85 (48%)	96 (29.7%)	<0.001
CKD	71 (14.2%)	35 (19.8%)	36 (11.1%)	0.008
**Clinical presentation**				
Abdominal/low back pain	495 (99%)	176 (99.4%)	319 (98.8%)	0.47
Pulsatile abdominal mass	438 (87.6%)	158 (89.3%)	280 (86.7%)	0.41
Hypovolemic shock	369 (73.8%)	160 (90.4%)	209 (64.7%)	<0.001
Collapse	240 (48%)	119 (67.2%)	121 (37.5%)	<0.001
Unconsciousness	95 (19%)	64 (36.2%)	31 (9.6%)	<0.001
Ventricular arrhythmia	37 (7.4%)	25 (14.1%)	12 (3.7%)	<0.001
Cardiac arrest	10 (2%)	9 (5.1%)	1 (0.3%)	<0.001
**Haemodynamics**				
SAP < 70 (mmHg)	119 (23.8%)	75 (42.4%)	44 (13.6%)	<0.001
DAP < 60 (mmHg)	293 (58.6%)	142 (80.2%)	151 (46.7%)	<0.001
MAP < 60 (mmHg)	187 (37.4%)	100 (56.5%)	87 (26.9%)	<0.001
**Laboratory**				
Haemoglobin < 78 (g/L)	118 (23.6%)	69 (39%)	49 (15.2%)	<0.001
Platelets < 145 (10^9^)	154 (30.8%)	93 (52.5%)	87 (26.9%)	<0.001
Creatinine > 145 (mmol/L)	159 (31.8%)	83 (46.9%)	76 (23.5%)	<0.001

BMI—body mass index, COPD—chronic obstructive pulmonary disease, CKD—chronic kidney disease, AAA—abdominal aortic aneurysm, SAP—systolic arterial pressure, DAP—diastolic arterial pressure, MAP—mean arterial pressure.

**Table 2:** Anatomical and procedural data in the whole study group (*n* = 500).

**Anatomic characteristics**	
AAA size (mm)	80 ± 16.8
AAA localization	
Infrarenal	409 (81.8%)
Juxtarenal	76 (15.2%)
Iliac	14 (3%)
**Procedural characteristics**	
Position of proximal aortic clamp	
Infrarenal	145 (29%)
Suprarenal	16 (3.2%)
Supraceliac	339 (67.8%)
Duration of proximal aortic clamping (min)	36.6 ± 24.5
Type of reconstruction	
None	18 (3.6%)
Tube graft interposition	262 (52.4%)
AII bypass	140 (28%)
AFF bypass	80 (16%)
Total operative time (min)	166.5 ± 50.6
Blood loss (ml)	3000 (2000–4500)

AAA—abdominal aortic aneurysm, AII—aortobiiliac, AFF—aortobifemoral.

**Table 3:** Postoperative complications (*n* = 500).

Acute kidney injury	70 (14%)
Pulmonary complications *	111 (22.2%)
Surgical reintervention	69 (13.8%)
Major bleeding requiring reintervention	38 (7.6%)
Lower limb ischaemia	17 (3.4%)
Stroke	16 (3.2%)
Acute coronary syndrome	31 (6.2%)
Ischaemic colitis	26 (5.2%)
Sepsis	7 (1.4%)
Wound infection	6 (1.2%)
Wound dehiscence	4 (0.8%)
Abdominal compartment syndrome	13 (2.6%)

*—Pulmonary complications—prolonged ventilation more than 72 h, pneumonia, atelectasis.

**Table 4:** Factors that influenced 30-day mortality.

	Univariate Analysis			Multivariate Analysis		
Variables	OR	95%CI	*p* Values	OR	95%CI	*p* Values
Age > 74	4.56	3.08–6.76	<0.001	4.01	2.43–6.26	<0.001
Unconsciousness	5.33	3.30–8.63	<0.001	2.21	1.11–4.40	0.024
Previous myocardial infarction	2.23	1.41–3.53	<0.001	2.35	1.19–4.63	0.014
Ventricular arrhythmia	4.26	2.08–8.71	<0.001	4.54	1.75–11.78	0.002
DAP < 60 mmHg	4.62	3.00–7.10	<0.001	2.32	1.17–4.62	0.016

DAP—diastolic blood pressure, OR—odds ratio, CI—confidence interval.

**Table 5:** Predictors of 30-day mortality.

Predictors	OR	Points
Age > 74	4.01	1
Unconsciousness	2.21	1
Previous myocardial infarction	2.35	1
Ventricular arrhythmia	4.54	1
DAP < 60 mmHg	2.32	1

DAP—diastolic blood pressure, OR—odds ratio.

**Table 6:** Distribution according to preoperative risk score.

Score	0	1	2	3	4	5
Deaths, *n* (%)	7 (7.4%)	22 (15.3%)	44 (34.9%)	58 (68.2%)	37 (88.1%)	9 (100%)
Number of patients	94	144	126	85	42	9

## Data Availability

The datasets generated during and/or analyzed during the current study are available from the corresponding author on reasonable request.

## References

[1] Hoornweg L.L., Storm-Versloot M.N., Ubbink D.T., Koelemay M.L., Legemate D.A., Balm R. (2008). Meta analysis on mortality of ruptured abdominal aortic aneurysms. Eur. J. Vasc. Endovasc. Surg..

[2] Kapma M.R., Dijksman L.M., Reimerink J.J., de Groof A.J., Zeebregts C.J., Wisselink W., Balm R., Dijkgraaf M.G., Vahl A.C. (2014). Cost-effectiveness and cost-utility of endovascular versus open repair of ruptured abdominal aortic aneurysm in the Amsterdam Acute Aneurysm Trial. Br. J. Surg..

[3] Powell J.T., Sweeting M.J., Thompson M.M., Ashleigh R., Bell R., Gomes M., Greenhalgh R.M., Grieve R., Heatley F., IMPROVE Trial Investigators (2014). Endovascular or open repair strategy for ruptured abdominal aortic aneurysm: 30 day outcomes from IMPROVE randomised trial. BMJ.

[4] Hoornweg L.L., Wisselink W., Vahl A., Balm R., Amsterdam Acute Aneurysm Trial Collaborators (2007). The Amsterdam Acute Aneurysm Trial: Suitability and application rate for endovascular repair of ruptured abdominal aortic aneurysms. Eur. J. Vasc. Endovasc. Surg..

[5] Park B.D., Azefor N., Huang C.C., Ricotta J.J. (2013). Trends in treatment of ruptured abdominal aortic aneurysm: Impact of endovascular repair and implications for future care. J. Am. Coll. Surg..

[6] Holt P.J., Karthikesalingam A., Poloniecki J.D., Hinchliffe R.J., Loftus I.M., Thompson M.M. (2010). Propensity scored analysis of outcomes after ruptured abdominal aortic aneurysm. Br. J. Surg..

[7] Reimerink J.J., van der Laan M.J., Koelemay M.J., Balm R., Legemate D.A. (2013). Systematic review and meta-analysis of population-based mortality from ruptured abdominal aortic aneurysm. Br. J. Surg..

[8] Acosta S., Ogren M., Bergqvist D., Lindblad B., Dencker M., Zdanowski Z. (2006). The Hardman index in patients operated on for ruptured abdominal aortic aneurysm: A systematic review. J. Vasc. Surg..

[9] Thompson P.C., Dalman R.L., Harris E.J., Chandra V., Lee J.T., Mell M.W. (2016). Predictive models for mortality after ruptured aortic aneurysm repair do not predict futility and are not useful for clinical decision making. J. Vasc. Surg..

[10] Wanhainen A., Verzini F., Van Herzeele I., Allaire E., Bown M., Cohnert T., Dick F., van Herwaarden J., Karkos C., Koelemay M. (2019). ESVS Guidelines Committee. Editor’s Choice—European Society for Vascular Surgery (ESVS) 2019 Clinical Practice Guidelines on the Management of Abdominal Aorto-iliac Artery Aneurysms. Eur. J. Vasc. Endovasc. Surg..

[11] Markovic M., Tomic I., Ilic N., Dragas M., Koncar I., Bukumiric Z., Sladojević M., Davidović L. (2016). The Rationale for Continuing Open Repair of Ruptured Abdominal Aortic Aneurysm. Ann. Vasc. Surg..

[12] Von Elm E., Altman D.G., Egger M., Pocock S.J., Gøtzsche P.C., Vandenbroucke J.P., STROBE Initiative (2008). The Strengthening the Reporting of Observational Studies in Epidemiology (STROBE) statement: Guidelines for reporting observational studies. J. Clin. Epidemiol..

[13] Samy A.K., Murray G., MacBain G. (1994). Glasgow aneurysm score. Cardiovasc. Surg..

[14] Hardman D.T., Fisher C.M., Patel M.I., Neale M., Chambers J., Lane R., Appleberg M. (1996). Ruptured abdominal aortic aneurysms: Who should be offered surgery?. J. Vasc. Surg..

[15] Chen J.C., Hildebrand H.D., Salvian A.J., Taylor D.C., Strandberg S., Myckatyn T.M., Hsiang Y.N. (1996). Predictors of death in nonruptured and ruptured abdominal aortic aneurysms. J. Vasc. Surg..

[16] Tambyraja A., Murie J., Chalmers R. (2007). Predictors of outcome after abdominal aortic aneurysm rupture: Edinburgh Ruptured Aneurysm Score. World J. Surg..

[17] Robinson W., Schanzer A., Li Y., Goodney P., Nolan B., Eslami M., Cronenwett J.L., Messina L.M. (2013). Derivation and validation of a practical risk score for prediction of mortality after open repair of ruptured abdominal aortic aneurysms in a U.S. regional cohort and comparison to existing scoring systems. J. Vasc. Surg..

[18] Wise E., Hocking K., Brophy C. (2015). Prediction of in-hospital mortality after ruptured abdominal aortic aneurysm repair using an artificial neural network. J. Vasc. Surg..

[19] Garland B.T., Danaher P.J., Desikan S., Tran N.T., Quiroga E., Singh N., Starnes B.W. (2018). Preoperative risk score for the prediction of mortality afrer repair of ruptured abdominal aortic aneurysms. J. Vasc. Surg..

[20] Meguid R.A., Brooke B.S., Perler B.A., Freischlag J.A. Impact of hospital teaching status on survival from ruptured abdominal aneurysm repair. Proceedings of the Annual Meeting of Eastern Vascular Society.

[21] Oyague K.S., Mubarak O.A., Nowak L.R., Gainer J.G., Rehring T.F., O’Brien M.M., Hollis H.W. (2015). Endovascular repair of ruptured and symptomatic abdominal aortic aneurysms using a structured protocol in a community teaching hospital. Ann. Vasc. Surg..

[22] Mayer D., Aeschbacher S., Pfammatter T. (2013). Complete replacement of open repair for ruptured abdominal aortic aneurysms by endovascular aneurysm repair. A two-center 14-year experience. J. Vasc. Surg..

[23] McPhee J., Eslami M.H., Arous E.J., Messina L.M., Schanzer A. (2009). Endovascular treatment of ruptured abdominal aortic aneurysms in the United States (2001–2006): A significant survival benefit over open repair is independently associated with increased institutional volume. J. Vasc. Surg..

[24] Tang T., Lindop M., Munday I., Quick C.R., Gaunt M.E., Varty K. (2003). A cost analysis of surgery for ruptured abdominal aortic aneurysm. Eur J. Vasc. Endovasc. Surg..

